# Spectrophotometric evaluation of dental bleaching under orthodontic bracket in enamel and dentin

**DOI:** 10.4317/jced.51168

**Published:** 2014-10-01

**Authors:** Nadia Lunardi, Americo-Bortolazzo Correr, Alessandra-Nara-Souza Rastelli, Débora-Alves-Nunes-Leite Lima, Rafael-Leonardo-Xediek Consani

**Affiliations:** 1 Postdoc student. Restorativa Dentistry Department, School of Dentistry, State University of Campinas UNICAMP, Piracicaba, Brazil; 2 Postdoc student. Dental Materials Department, School of Dentistry, State University of Campinas UNICAMP, Piracicaba, Brazil; 3 Assisstent Professor. Restorative Dentistry Department, School of Dentistry, State University of São Paulo – UNESP, Araraquara, Brazil; 4 Assistent Professor. Restorative Dentistry Department, School of Dentistry, State University of Campinas UNICAMP, Piracicaba, Brazi; 5 Assistent Professor. Prostetic Department, School of Dentistry, State University of Campinas UNICAMP, Piracicaba, Brazil

## Abstract

Aware of the diffusion capacity of bleaching in the dental tissues, many orthodontists are subjecting their patients to dental bleaching during orthodontic treatment for esthetic purposes or to anticipate the exchange of esthetic restorations after the orthodontic treatment. For this purpose specific products have been developed in pre-loaded whitening trays designed to fit over and around brackets and wires, with clinical efficacy proven. Objective: The objective of this study was to evaluate, through spectrophotometric reflectance, the effectiveness of dental bleaching under orthodontic bracket. Material and Methods: Thirty-two bovine incisors crown blocks of 8 mm x 8 mm height lengths were used. Staining of tooth blocks with black tea was performed for six days. They were distributed randomly into 4 groups (1-home bleaching with bracket, 2- home bleaching without bracket, 3- office bleaching with bracket, 4 office bleaching without bracket). The color evaluation was performed (CIE L * a * b *) using color reflectance spectrophotometer. Metal brackets were bonded in groups 1 and 3. The groups 1 and 2 samples were subjected to the carbamide peroxide at 15%, 4 hours daily for 21 days. Groups 3 and 4 were subjected to 3 in-office bleaching treatment sessions, hydrogen peroxide 38%. After removal of the brackets, the second color evaluation was performed in tooth block, difference between the area under the bracket and around it, and after 7 days to verified color stability. Data analysis was performed using the paired t-test and two-way variance analysis and Tukey’s. Results: The home bleaching technique proved to be more effective compared to the office bleaching. There was a significant difference between the margin and center color values of the specimens that were subjected to bracket bonding. Conclusions: The bracket bond presence affected the effectiveness of both the home and office bleaching treatments.

** Key words:**Tooth bleaching, spectrophotometry, orthodontics.

## Introduction

The color change of teeth during orthodontic treatment has been proven by studies such as Trakyal? *et al.* [2009], Karamouzos *et al.* [2010], Çörekçi *et al.* [2010] (1-3). This undesirable effect may occur due to staining of the enamel and the resin material used for the bonding of brackets. In enamel, the color change may be the result of demineralization (4) or the direct absorption of food dye (3,5). The staining of the resin material is associated with the color instability of the polymer (6).

The dental bleaching mechanism occurs through a redox reaction where hydrogen peroxide reduces the organic pigments impregnated in enamel and dentin, allowing its elimination. In contact with the dental enamel, hydrogen peroxide releases unstable oxygen that joins other substances that are free or weakly bound to a particular substrate, thereby achieving stabilization again. This is made possible by the large electronegativity of oxygen, which gives it enormous power of reaction. Therefore, the ion oxygen reacts with tooth-staining molecules, breaking them, and generating smaller molecules or single bonds that are lighter and can be eliminated (7).

Dental bleaching can be performed using one of two techniques: home, also known as self-administration, and office (8). Home bleaching agents are administered in low concentration in a flexible mold used daily for long periods of exposure supervised by a dentist [an average of four hours/day]. Office bleaching uses a high concentration of bleaching agents for short periods of exposure [on average of 45 min/session] (7-8).

Aware of the diffusion capacity of bleaching in the dental tissues, many orthodontists are subjecting their patients to dental bleaching during orthodontic treatment for esthetic purposes or to anticipate the exchange of esthetic restorations after the orthodontic treatment (9). For this purpose specific products have been developed in pre-loaded whitening trays designed to fit over and around brackets and wires. About this question only one clinical research was found, which proved its effectiveness (10).

Thus, the scientific inquiry of this study is about the effectiveness of the bleaching agent action under the resin tags, in order to know whether this technique would be effective in whitening teeth with color change prior to orthodontic treatment, in order to really change the initial color of the tooth, and not only to clear staining occurred during ortodontic treatment.

## Material and Methods

Thirty-two bovine incisors were selected for this study and stored in a 0.1% thymol solution after cleaning. Tooth crown blocks of 8 mm x 8 mm height lengths were cut with an Isomet 1000® [Buehler, Lake Bluff, Illinois, USA] precision cutter, and flattened with silicon carbide paper to obtain a standardized thickness of 1 mm-1.5 mm of enamel and dentin. The sample size [8 x 8mm] was defined in order to have enough surface enamel around the bracket to be possible measure the difference of the around bracket area and below bracket area. Staining of tooth blocks with black tea was performed for six days (11,12). After prophylaxis they were evaluated for staining homogeneity for standardization, and distributed randomly into 4 groups [1-home bleaching with bracket, 2- home bleaching without bracket, 3- office bleaching with bracket, 4 office bleaching without bracket]

**Figure 1:**
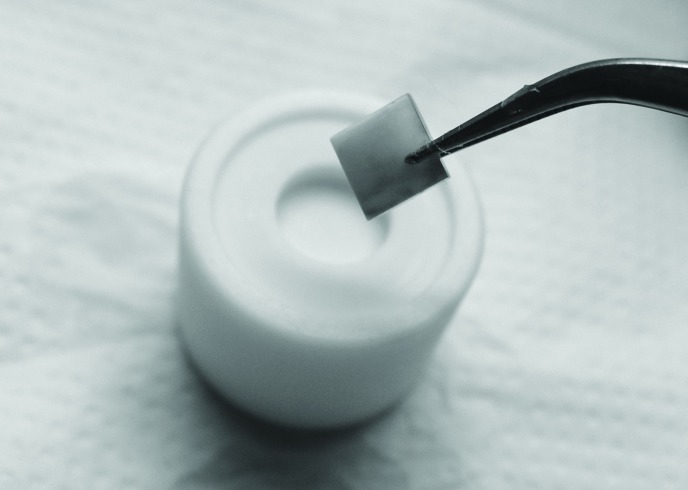
The specimens placed in a Teflon sample holder made for the tooth block size.

Each specimen was marked with a round bur on one side to standardize the sample positioning during the color evaluation. The specimens were placed in a Teflon sample holder made for the tooth block size (Fig. 1). The evaluation was performed using a Minolta CM 700D color reflectance spectrophotometer [Minolta Co. Ltd., Tokyo, Japan] in ambient light in a standard light booth [GTI Graphic Technology Inc., Newburgh, NY, USA]. The spectrophotometer was calibrated according to the manufacturer’s instructions using SAV [small area view: 5 mm reading area].

To perform the reading, it was necessary to the perfect the coupling of the reading end of the spectrophotometer to the specimen holder, preventing the entry of external light. A light cabinet regulated in daylight was used to standardize the incident light around the specimen holder. After enamel and dentin reading was done. The values obtained were subsequently exported to a software program for color verification [Color On, Konica Minolta Sensing Americas, Ramsey, New Jersey, USA]. For the color evaluation, CIE L * a * b * was used; this model was proposed by the Commission Internationale de l’Éclairage [CIE], the standardization organization for color and appearance in defined areas. The specimens were divided into 4 groups [n = 8] by the random selection of the numbers 1-32 in accordance with treatment [bonding of the dental bracket and bleaching].

Metal brackets [Agile Mini, Absil 3M Brazil, São José do Rio Preto, Brazil] were bonded in groups 1 and 3 according to the bond material manufacturer’s instructions [Transbond XT 3M Unitek, Monrovia, California, USA] with controlled humidity [50%] and temperature [23?C]. After bonding, the specimens were stored in artificial saliva, changed daily, at 37?C for 24 hours.

Seventy-two hours after dyeing and 24 hours after bonding, the groups 1 and 2 samples were subjected to the Opalescence PF Regular, carbamide peroxide at 15%, [Ultradent Products, Inc. South Jordan, USA] 4 hours daily for 21 days. To prevent dehydration, the specimen container was placed without a lid in a closed larger container with water to maintain 100% humidity and stored in an oven [37?C] for four hours to simulate the humidity conditions of the mouth. Groups 3 and 4 were subjected to 3 in-office bleaching treatment sessions Opalescence Boost PF Regular, hydrogen peroxide 38%, [Ultradent Products, Inc. South Jordan, USA] was used according to the manufacturer’s recommendations: 3 sessions, 1 session per week, 45 min/session. After bleaching, the specimens were washed with for complete removal of the bleaching agent and stored in artificial saliva.

The brackets were removed manually with bracket remover pliers [OrthoSource, Porto Alegre, RS, Brazil]. Removal of the residual resin was performed with aluminum oxide tips [Shofu Dental Coporation, Menlo Park, California, USA], and Enhance finishing [Dentisply, Petrópolis, RJ, Brazil] at low speed. To return the shine to the enamel, the specimens were polished using fine sandpaper to finish granulation 1200 for 15 seconds. Verification of the presence of residual resin was performed with a magnifying glass and stereoscopic explorer.

**Figure 2:**
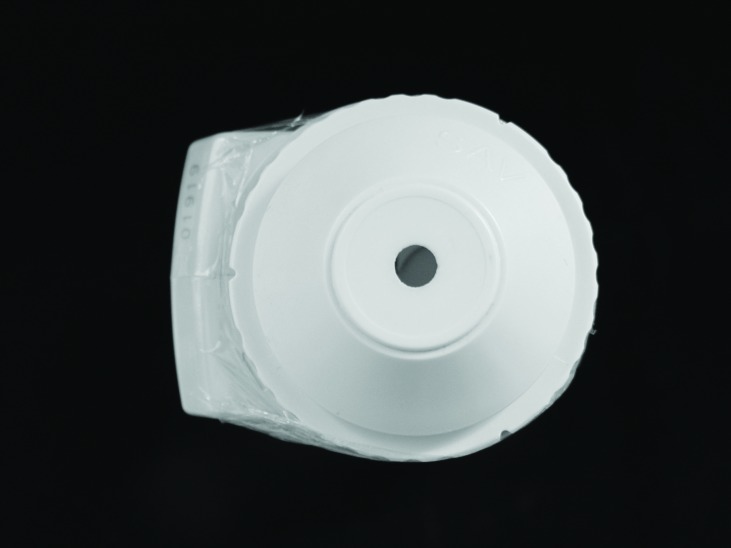
Illumination/reading area of the SAV of 5 mm.

**Figure 3:**
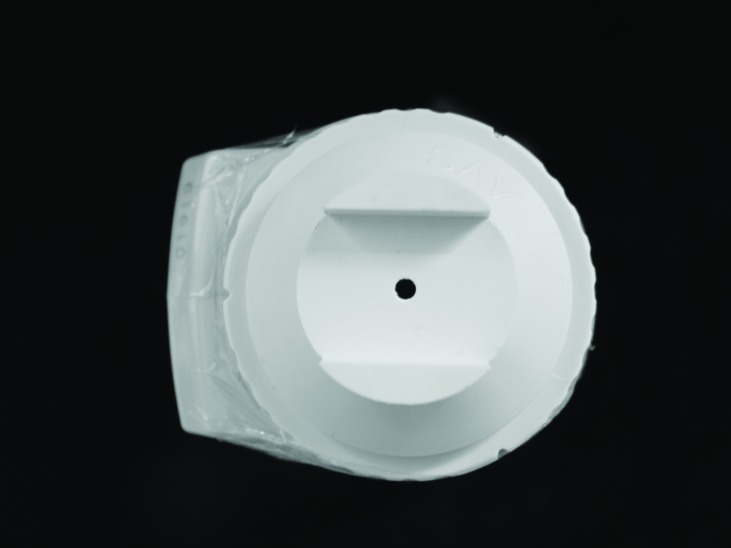
Illumination/reading area of the SAV of 3 mm.

The specimens were again subjected to a spectrophotometer color reading, similar to the first reading. Seven days after the treatment third read was performed to evaluate the bleaching stability. To accurately assess the color change in the region under the bracket, a PVC-coupled device with a 3 mm aperture for passing light was used, perfectly adapted to the tip of the apparatus by reducing the illumination/reading area of the SAV of 5 to 3 mm (Figs. 2,3). Thus, the spectrophotometer color readings were taken in the center [metal bracket bonded] and the edge of specimen.

To color differences measure [?E] was necessary to use the formulas recommended by the CIE lab method: ?E = ? [?L]2 + [?a]2 + [?b]2 where ?L = L1[final] - L0 [initial], ?a = a1[final] - a0 [initial] , ?b = b1[final] - b0 [initial] to evaluate the bleaching treatment in dental blocks; and ?L = L1[edge] - L0[center], ?a = a1[edge] - a0[center] , ?b = b1[edge] - b0[center] for evaluation of color difference in each dental block under and around bonded bracket in the end of treatment.

To verify the normality of the data, for both enamel and dentin, we performed the Kolmogorov-Smirnov test. After verified the normal distribution of data, tests were conducted with a significance level of 5%: paired t-test was used to verify the effectiveness of the bleaching treatment [initial and final] and its stability after 7 days [late and 7 days] for each coordinated color; and two-factor analysis of variance and Tukey’s test were performed for each substrate [enamel and dentin] and coordinated color.

## Results

In assessing bleaching treatment effectiveness [initial-final], there was a significant difference in all factors evaluated [L, a, b]; except dentin coordinate b for office bleaching without bracket ( Table 1). Color stability evaluation showed lack of stability for factor L in enamel, and reducing the value in home bleaching and an increase in office ( Table 2). In evaluating delta E there was no statistical difference when realized with SAV 5mm reading area. However, with the reduction reading area [SAV 3mm] was possible to observe a color difference under the bracket [center] compared the area around it [edge], demonstrating reduced efficiency of bleaching below the bracket ( Table 3, Table 4).

**Table 1:**
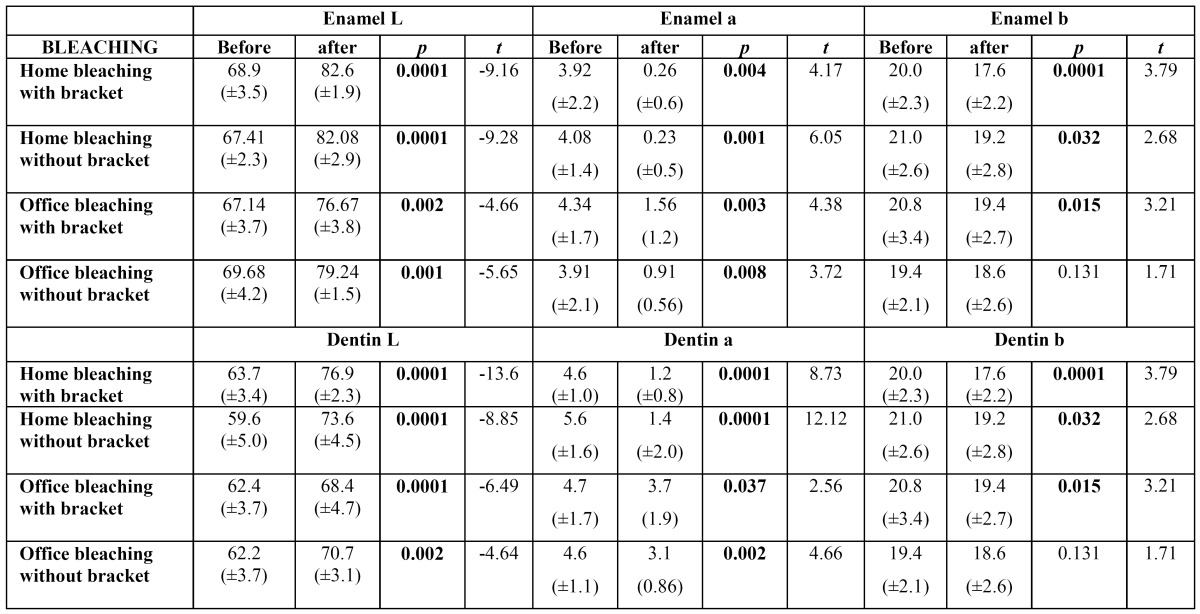
Mean and standard deviation for coordinated L, a e b before and after bleaching in enamel and dentin.

**Table 2:**
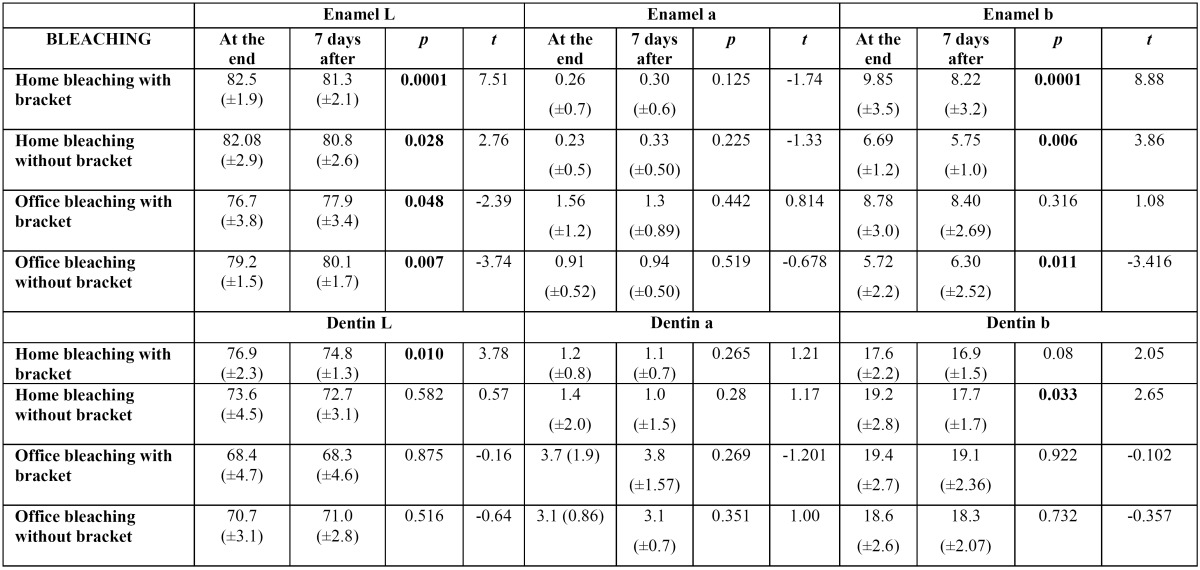
Mean and standard deviation for coordinated L, a e b end of the bleaching and 7 days after the end of bleaching in enamel and dentin.

**Table 3:**
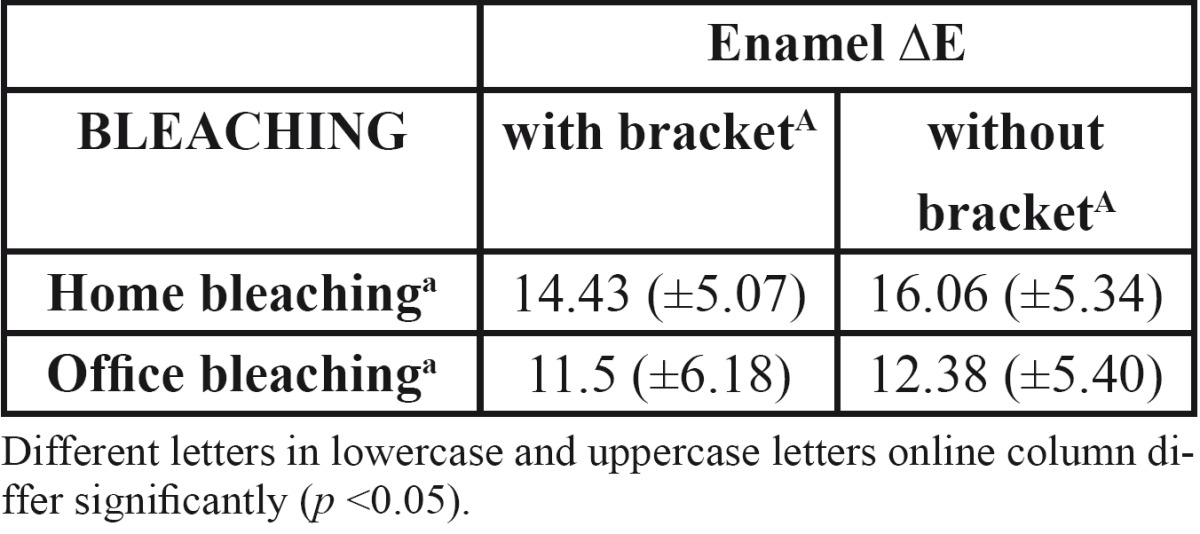
Mean and standard deviation for the ?E parameter reading for enamel after bleaching treatment.

**Table 4:**
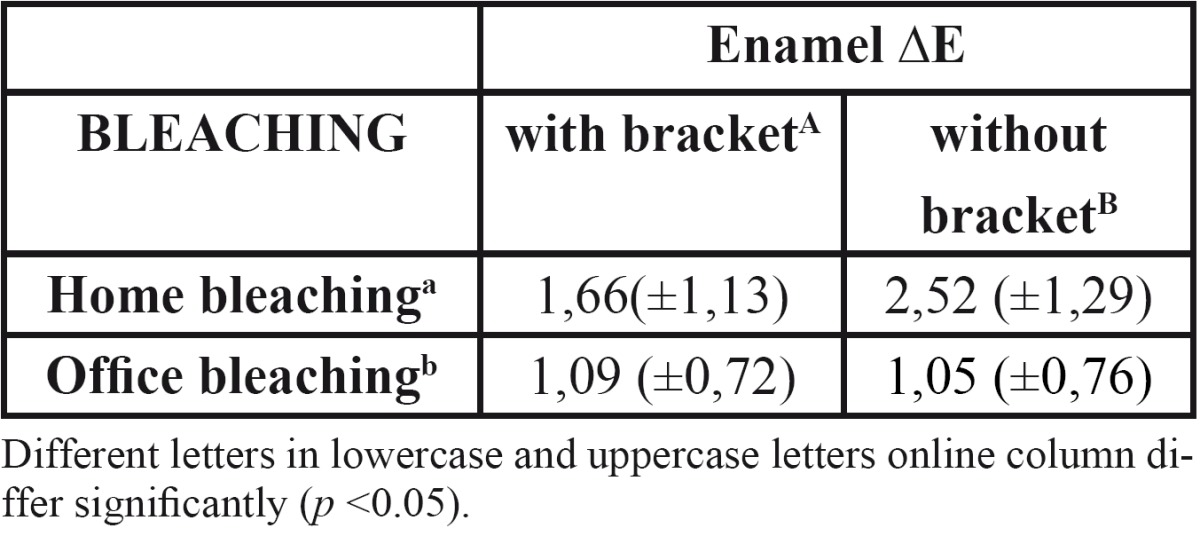
Mean and standard deviation for ?E enamel in the center and edge of the specimen, with reading area (SAV) 3mm.

## Discussion

The enamel acts as a semipermeable membrane permitting the passage of water, oral fluid (13) and free radicals, and the dentinal tubules formed around the extension odontoblastic, have communications with one another throughout their length. The degree of permeability of these tissues can surely act as the bleaching more deeply affecting the dentin, though this appears to be a gradual process, and is surely prevented by the resin tags present in enamel, as demonstrated in this study.

Using spectrophotometer with SAV 5mm opening demonstrated the effectiveness of bleaching treatment in almost all groups, however, it was possible to observe clearly a darker region of bonding, showing the ineffectiveness of bleaching in this region, which was possibly evidenced by staining of the teeth prior to bonding of brackets. The obvious difference in color between the center and the edges of the block tooth not being detected by the spectrophotometer, led us to question the extent of reading, which possibly would mask this result.

This study proved that the presence of the bracket bond negatively affected the effectiveness of home and office bleaching treatment, and that home bleaching is more efficient than the in-office technique. The use of standardized flat samples, staining homogeneity and the same polishing degree contributed to the accuracy of evaluation. The use of bovine teeth allowed standardization of the specimen size, planning, and the thickness of enamel and dentin, which would obviously be very difficult in human teeth.

In this study, only when the spectrophotometer reading diameter was reduced from 5 mm to 3 mm was it possible to verify color variation in the same sample [center and edge] ( Table 4). It is possible that a larger opening was unable to distinguish the color variation ( Table 3) occurring in the center of the specimen since the amount of tooth bleached without the presence of the bracket was much larger than the portion below the tooth attachment.

The results showed that the bonding of the orthodontic bracket and contact ausence enamel-bleaching gel affected the outcome of the dental bleaching treatment, as the bleach was unable to penetrate evenly throughout the specimen, resulting in a poorly lightened area under the bracket. This result corroborates the study of Hintz, Bradle, Eliades [2001] (14), who observed the difficulty of the bleaching agent’s spreading where the bracket had been bonded and taken off. According to these authors, it is possible that tags of the remaining resin material hindered the diffusion and action of the bleaching agent.

The ineffectiveness of the bleaching agent under a bonded orthodontic bracket, it can be suggest that the effectiveness demonstrated by Jadad, Montoya, Arana, Gordillo, Palo, Loguercio [2011] (11) was due to the failure to distinguish color variation in human teeth caused by the greatest diameter opening read/brightness of the spectrophotometer. Another possibility would be that, as it is demonstrable that orthodontic appliances (1-3) generate a color change in the teeth, it is possible that a portion of the tooth below the bracket will remain the original color, whereas the adhesion prevents the impregnation of dyes. Bleaching in the presence of brackets could cause the tooth to return to the initial color, contributing to color homogeneity of the tooth crown.

In this study, the home dental bleaching technique proved to be superior to the in-office bleaching technique in the ?E in dentin. In a literature review, Joiner [2006] (8) described many comparisons of time, concentration, and type of bleaching gel and showed conflicting results regarding home and in-office techniques, and he cited the extensive diversity of bleaching, concentrations, treatment, and research methods as a reason. However, the results of this study agree with some of the articles that evaluated the two types of dental bleaching procedures (15-18). It is possible that this result is based on the justification given by Dietsch, Campanile, Holz, Meyer [2006] (16), who reported that a home bleaching technique with carbamide peroxide presented greater efficiency in clear deeper structures such as dentin due to the continuous and longer time release of hydrogen peroxide. In contact with water, carbamide peroxide results in urea and hydrogen peroxide. The urea will yield ammonia and carbon dioxide, thus helping to maintain an alkaline pH, which enhances the action of the bleaching. The carbopol, a thickener present in carbamide peroxide bleaching gel, is a polyacrylic buffered acid that also retards the degradation of carbamide peroxide, allowing the release of hydrogen peroxide more gradually, making the gel effective for a longer period (6). As the enamel works as a semipermeable membrane allowing the passage of water, oral fluid (19), and free radicals, and dentin has tubules that connect with each other throughout their length, the degree of permeability of these tissues allows the bleaching treatment to act deeply, reaching the dentin. However, this process seems to be gradual and will certainly be prevented by resin tags that are present in the bond bracket enamel (20).

Bovine incisor physical-chemical characteristics do not differ considerably from human dentin. The bovine dentin tubule diameter is 3-5µm with 20,000 tubules/mm2, and human dentin, in the outer layers near the enamel, is 0.5-1.2 µm with 10,000-25,000 tubules/mm2 (19). This is an important factor when the peroxide diffusion is quantified. However, this was not evaluated in the present study. According to Attia, Aguia, Mathias [2009] (21), in an in vitro study, bovine and human dental substrates behave similarly during the bleaching process, probably due to their morphological similarity.

Concerning color coordinates, Trakyali, Özdemir, Arun [2009] (3) and Karpinia, Magnusson, Sagel, Zhou, Gerlach [2002] (22) agreed in reporting that the ?L would be the most significant parameter in assessing dental bleaching, whereas the human eye detects changes in brightness [?L] more easily than the other color parameters [?a ?b]. Karpinia, Magnusson, Sagel, Zhou, Gerlach [2002] (22) observed significant improvement in yellowness [?b] and lightness [?L] after the bleaching procedure. However, Carvalho, Robazza, Lage-Marques [2002] (23) found a significant difference in ?L and no significant difference in the overall evaluation of color ?E.

This study revealed that, besides the significant change of lightness [L*] in the enamel and dentin, it was verified that there was a reduction of red, reaching more neutral colors [white and gray] for the home treatment [a*] and a reduction of yellow that was more significant in groups without brackets [b*].

In relation to color stability after 7 days, this work demonstrated that the home bleaching treatment was less stable, showing changes in enamel and dentin; however, the office treatment showed greater stability and even bleaching continuation for 7 days after the final treatment, observed by the increase of the coordinate L value. The high concentration of hydrogen peroxide inside the tooth structure after in-office bleaching makes it continue to act after the treatment, giving a false impression of stability. These results corroborate those Wiegand, Vollmer, Foitzik, Attin, Attin [2005] (24) that confirmed the lack of stability of dental bleaching.

Also, regarding color stability, substrate dentin proved to be more stable compared to enamel because of coordinate a*. In this study, there was discoloration in the enamel but not in the dentin, which contradicts Wiegand, Vollmer, Foitzik, Attin, Attin [2005] (24), who concluded that bleached tooth color was affected more by the color change of the dentin.

## Conclusions

It was evident that the bracket bond presence negatively affected the effectiveness of both the home and in-office bleaching treatments.
